# ChIPpeakAnno: a Bioconductor package to annotate ChIP-seq and ChIP-chip data

**DOI:** 10.1186/1471-2105-11-237

**Published:** 2010-05-11

**Authors:** Lihua J Zhu, Claude Gazin, Nathan D Lawson, Hervé Pagès, Simon M Lin, David S Lapointe, Michael R Green

**Affiliations:** 1 Program in Gene Function and Expression, University of Massachusetts Medical School, Worcester, Massachusetts 01605, USA; 2 Program in Molecular Medicine, University of Massachusetts Medical School, Worcester, Massachusetts 01605, USA; 3 UMR217 CNRS/CEA, iRCM-CEA, Evry, Ile de France 91057, France; 4 Program in Computational Biology, Fred Hutchinson Cancer Research Center, Seattle, Washington, 98109-1024, USA; 5 The Biomedical Informatics Center, Northwestern University, Chicago IL 60611, USA; 6 Information Services, University of Massachusetts Medical School, Worcester, Massachusetts 01605, USA

## Abstract

**Background:**

Chromatin immunoprecipitation (ChIP) followed by high-throughput sequencing (ChIP-seq) or ChIP followed by genome tiling array analysis (ChIP-chip) have become standard technologies for genome-wide identification of DNA-binding protein target sites. A number of algorithms have been developed in parallel that allow identification of binding sites from ChIP-seq or ChIP-chip datasets and subsequent visualization in the University of California Santa Cruz (UCSC) Genome Browser as custom annotation tracks. However, summarizing these tracks can be a daunting task, particularly if there are a large number of binding sites or the binding sites are distributed widely across the genome.

**Results:**

We have developed *ChIPpeakAnno *as a Bioconductor package within the statistical programming environment R to facilitate batch annotation of enriched peaks identified from ChIP-seq, ChIP-chip, cap analysis of gene expression (CAGE) or any experiments resulting in a large number of enriched genomic regions. The binding sites annotated with *ChIPpeakAnno *can be viewed easily as a table, a pie chart or plotted in histogram form, i.e., the distribution of distances to the nearest genes for each set of peaks. In addition, we have implemented functionalities for determining the significance of overlap between replicates or binding sites among transcription factors within a complex, and for drawing Venn diagrams to visualize the extent of the overlap between replicates. Furthermore, the package includes functionalities to retrieve sequences flanking putative binding sites for PCR amplification, cloning, or motif discovery, and to identify Gene Ontology (GO) terms associated with adjacent genes.

**Conclusions:**

*ChIPpeakAnno *enables batch annotation of the binding sites identified from ChIP-seq, ChIP-chip, CAGE or any technology that results in a large number of enriched genomic regions within the statistical programming environment R. Allowing users to pass their own annotation data such as a different Chromatin immunoprecipitation (ChIP) preparation and a dataset from literature, or existing annotation packages, such as *GenomicFeatures *and *BSgenom*e, provides flexibility. Tight integration to the *biomaRt *package enables up-to-date annotation retrieval from the BioMart database.

## Background

ChIP followed by high-throughput sequencing (ChIP-seq) and ChIP followed by genome tiling array analysis (ChIP-chip) have become standard high-throughput technologies for genome-wide identification of DNA-binding protein target sites [1-4]. A number of algorithms and tools have been developed for analyzing the large datasets generated by ChIP-chip (reviewed in [4]) and ChIP-seq experiments [1,5-10]. The output from such algorithms is typically presented as a list of binding sites (also referred to as peaks) that are significantly enriched in the ChIP sample compared to the control sample(s). The identified binding sites are usually converted to a format, such as BED or Wiggle (WIG), that can be uploaded to the UCSC Genome Browser, an open-access, web-based, up-to-date source for genome sequence data integrated with a large collection of related annotations [11]. This resource allows the user to build a custom annotation track to view the proximity of the DNA-binding sites to various genomic features such as genes, exons, transcription start sites and conserved elements. However, searching the UCSC Genome Browser can be a daunting task for the user, particularly if there are a large number of binding sites or the binding sites are distributed widely across the genome.

Several useful web applications have been developed for managing and annotating ChIP-chip data [12-14] and ChIP-seq data [14]. However, there is a need for technology platform-independent and genome-independent batch annotation tools. Here we describe a Bioconductor package called *ChIPpeakAnno *that facilitates batch annotation, using a variety of annotation sources, of binding sites identified by any technologies which result in large number of enriched genomic regions, such as ChIP- chip, ChIP-seq and CAGE. *ChIPpeakAnno *leverages the statistical environment R/Bioconductor with various sources of annotations, such as Ensembl, the UCSC genome database and others. In addition, users have the flexibility to label enriched regions with any annotation of interest such as a dataset from the literature. This package is available from Bioconductor, an open source and open development software project specializing in biological data analysis and integration based on R, a system for statistical computation and graphics [15,16]. Bioconductor tools are distributed as separate but interoperable packages, each specializing in different areas of biological data analysis, such as the *limma *package for analyzing microarray data [17] and the *biomaRt *package for retrieving genomic annotation from the federated query system BioMart Ensembl [11,18,19]. The *ChIPpeakAnno *package contains various functionalities to batch-annotate enriched regions identified from ChIP-seq, ChIP-chip or CAGE experiments.

*ChIPpeakAnno *emphasizes flexibility, integration and ease of use. Users are supplied with functionalities for annotating peaks from ChIP-seq, ChIP-chip, CAGE or any experiment resulting in a list of chromosome coordinates with any annotation track users are interested in. Even though some of the functionalities such as the retrieval of neighbouring sequences for a set of peaks are available in other software programs, the complete set of tools and the flexibility offered by *ChIPpeakAnno *are not available in any other software. The main differentiating point from *CEAS, CisGenome *and other software is that *ChIPpeakAnno *enables comparison between a set of peaks with any annotation feature objects, for example comparing to CpG islands, to conserved elements (or other annotated features not captured by *CEAS *http://ceas.cbi.pku.edu.cn/submit.htm or *CisGenome *http://www.biostat.jhsph.edu/~hji/cisgenome/index.htm) (survey results) or comparing two sets of peaks between replicates (personal communication with Ivan Gregoretti at NIH) or transcription factors within a complex (unpublished data). In addition, unlike *ChIPpeakAnno*, *CEAS *or *CisGenome *does not have overlapping significance testing or Gene Ontology (GO) enrichment testing implemented. GO is a system for describing the molecular function, biological process and cellular compartmentalization of gene products [20]. Another main advantage of *ChIPpeakAnno *is the ability/flexibility to plug in with other annotation packages, such as *biomaRt *[17] and *GO.db*, ChIP-chip analysis packages such as *Ringo *[21] and *ACME *[22], other fast moving deep-sequencing analysis capabilities and infrastructure (Table 1) such as *ShortRead *[23], *DEGseq *[24], edgeR [25], *BayesPeak *[26], *chipseq, ChIPseqR, Rolexa *[27], *BSgenome, IRanges, Biostrings, rtracklayer *[28], GenomeGraphs [29] and statistical analysis tools such as *multtest and limma *[17] in Bioconductor (survey results).

**Table 1:** An overview of Bioconductor packages for analyzing high-throughput sequencing data.

Package	Classification	Functionalities
*ShortRead*	Input/Output QA Filtering	Supplies methods for reading, quality assessment (QA) and basic manipulation of high-throughput sequencing data.

*Rolexa*	Base Calling QA	Supports probabilistic base calling, quality checks and diagnostic plots for Solexa sequencing data.

*IRanges*	Infrastructure Ranged-based algorithm	Provides infrastructure for representing and manipulating sets of integer ranges, and implements algorithms for range-based calculations such as intersect, union, disjoint, overlap and coverage.

*BSgenome*	Whole Genome Annotation Data	Supplies infrastructure for efficiently representing, accessing and analyzing whole genome.

*Biostrings*	String manipulation	Implements functions for pattern matching, sequence alignment and string manipulation

*rtracklayer*	Visualization	Provides an interface between R and genome browsers and implements functions to import, create, export, and display track data by linking R with existing genome browsers.

*GenomeGraphs*		Integrates Ensembl annotation obtained using the biomaRt package and the grid graphic package to facilitate visualization, plotting and analysis of a diverse genomic datasets.

*ChIPpeakAnno*	Annotation Plotting Overlap test Enrichment test	Implements a common annotation workflow for ChIP-seq data such as finding nearest or overlapping features and obtaining enriched GO terms. In addition, it contains functions for determining the significance of the overlap and visualizing the overlap as a Venn diagram among different datasets.

*Genominator*	Annotation Summarization	Offers an interface for storing and retrieving genomic data in SQLite database.

*ChIPsim*	Simulation of ChIP-seq experiments	Provides a framework for the simulation of ChIP-seq experiments such as nucleosome positioning and transcription factor binding sites.

chipseq*	Analysis of ChIP-seq data	Implements basic workflow for analyzing ChIP-seq experiments, including functions to extend reads, calculating genomic coverage, and identifying peaks.
		
CSAR*		Contributes methods to normalize the count data and detect protein-bound genomic regions with controlled false discovery rate through random permutation. Models the sequence counts as poison distribution.
		
BayesPeak*		Identifies peaks using hidden Markov models and Bayesian statistical methodology. Models the sequence counts as the negative binomial distribution.

*ChIPseqR*	Analysis of nucleosome ChIP-seq data	Furnishes functions to analyze nucleosome ChIP-seq data and may be adapted to handle other types of ChIP-seq experiments.

*edgeR*	Analysis of RNA-seq data	Provides statistical routines for determining differential expression in count-based expression data such as RNA-seq, SAGE and CAGE. The RNA-seq data are modelled as the negative binomial distribution and applied with empirical Bays procedure.
		
*DEGseq*		Implements functions for identifying differentially expressed genes from RNA-seq data by modelling the RNA-seq data as the binomial distribution.
		
*baySeq*		Contains methods to determine differential expression in count based expression data with more complex experimental designs using Bayesian methods.
		
DESeq*		Provides functions for identifying differentially expressed genes from RNA-seq data by modelling the RNA-seq data as the negative binomial distribution.

goseq*	Enrichment testing of RNA-seq data	GO enrichment testing for RNA-seq data.

*Available in BioC 2.6 in R 2.11.0.

Usability is the top priority for *ChIPpeakAnno*. Once the package is loaded, one line of code (*annotatePeakInBatch*) enables users to find nearest or overlapping features such as gene, exon, miRNA, 5' utr, 3' utr, peaks from another dataset or any annotation track of interest. Users are also provided with the flexibility and functionality to get the annotation on the fly (*getAnnotation*). Two lines of code (*getEnrichedGO*) allow users to find enriched gene ontology terms. One line of code (*makeVennDiagram*) allows users to draw a Venn diagram and provide a p-value for determining the significance of the overlapping between datasets. Repeated calling of function *findOverlappingPeaks *enables users to find the overlapping among peaks from several different experiments, which will help users to determine how peaks from different replicates overlap and how peaks from different transcription factors within a complex overlap.

## Implementation

*ChIPpeakAnno *implements a common annotation workflow for ChIP-seq or ChIP-chip data in R, a system for statistical computation and graphics [15,16]. To promote component reuse and compatibility among Bioconductor packages, *ChIPpeakAnno *utilizes the *IRanges *package and represents the peak list as *RangedData *to efficiently find the nearest or overlapping gene, exon, 5' utr, 3' utr, microRNA (miRNA) or other custom feature(s) supplied by the user, such as the most conserved non-coding element, CpG island or transcription factor binding sites. All peak-calling software produces a file containing at least a list of chromosome coordinates that is all *ChIPpeakAnno *package needs. Both BED http://genome.ucsc.edu/FAQ/FAQformat#format1 and GFF (General Feature Format, http://genome.ucsc.edu/FAQ/FAQformat#format3) are common file formats that provide a flexible way to define peaks or annotations as data lines. Therefore, conversion functions *BED2RangedData *and *GFF2RangedData *were implemented for converting these data formats to a *RangedData *object. Since the genome annotations are updated periodically/frequently, we have leveraged the *biomaRt *package from Bioconductor to enable retrieval of annotation data on the fly from Ensembl. For fast access, transcription start sites from common genomes such as *TSS.human.NCBI36, TSS.human.GRCh37, ExonPlusUtr.human.GRCh37, TSS.mouse.NCBIM37, TSS.rat.RGSC3.4 and TSS.zebrafish.Zv8 *were included as pre-built annotation data packages. Users also have the flexibility to pass annotation data from the *GenomicFeatures *package as well as their own annotation data, such as a list of binding sites from other transcription factors, a different ChIP preparation or a different peak-calling algorithm. We have also utilized the *BSgenome *package to implement functions that allow retrieval of flanking sequences associated with peaks of interest. This facilitates subsequent PCR amplification, cloning and/or motif discovery using algorithms such as MEME [3,30]. To ascertain whether the identified peaks are enriched around genes with certain GO terms, we have implemented a GO enrichment test. This test applies the hypergeometric test *phyper *in R and integrates with GO annotation from the *GO.db *package, species-specific GO annotation packages such as *org.Hs.eg.db *and multiplicity adjustment functions from the *multtest *package in Bioconductor. GO annotation packages are updated per release that corresponds to twice a year. Binding sites annotated with *ChIPpeakAnno *can be exported as an Excel file to allow easy sorting and statistical analysis of larger lists of peaks. Alternatively, the distribution of peaks relative to genomic features of interest (e.g., transcription start site or exon start site) can be easily plotted for viewing as pie chart or histograms. In addition, we have implemented functionalities using hypergeometric test for determining the significance of overlap between replicate experiments, different peak-calling algorithms or binding sites among transcription factors within a complex, and for drawing Venn diagrams to visualize the extent of the overlap between replicates.

## Results

### Example 1: Finding the nearest gene and the distance to the transcription start site of the nearest gene

The output from ChIP-seq or ChIP-chip analysis is a list of binding sites (as chromosome locations) that are significantly enriched in the ChIP sample(s) compared with the corresponding control sample(s). The example below details how to find the nearest gene and the distance to the transcription start site (TSS) of the nearest gene in the human genome for a list of binding sites (named *myPeakList*) of type *RangedData*. The distance is calculated as the distance between the start of the binding site and the TSS, which is the gene start for genes located on the forward strand and the gene end for genes located on the reverse strand.

The first step is to load the *ChIPpeakAnno *package, an example dataset and an annotation dataset. In this example, the example dataset contains putative STAT1-binding regions identified in un-stimulated cells [2], and the annotation dataset contains the TSS coordinates and strand information from human GRCh37.

>library(ChIPpeakAnno)

>data(myPeakList)

*>data(TSS.human*.GRCh37)

In the next step, the function a *nnotatePeakInBatch *is called to find the gene with nearest TSS or overlapping gene that is not the nearest TSS and corresponding distance for the list of binding regions. Sometimes, a peak is within a gene but far from the gene's TSS. Setting the parameter *output *to "both" outputs both the genes with nearest TSS and the overlapping gene. The parameter *maxgap *sets the maximum gap to be considered as overlapping. The parameter *multiple *indicates whether multiple overlapping features should be returned for one peak.

>annotatedPeak = annotatePeakInBatch (myPeakList, AnnotationData = TSS.human.GRCh37, output="both", multiple = F, maxgap = 0)

The annotated peaks can be saved as an Excel file for biologists to view easily.

>write.table(as.data.frame(annotatedPeak), file = "annotatedPeakList.xls", sep = "\t", row.names = FALSE)

Plotting the distribution of the peaks relative to the TSS gives a birds-eye view of the peak distribution relative to the genomic features of interest.

*>y = annotatedPeak$distancetoFeature [!is.na(annotatedPeak$distancetoFeature) *&*annotatedPeak$fromOverlappingOrNearest == "NearestStart"]*

>hist(y, xlab = "Distance To Nearest TSS", main = "", breaks = 1000, xlim = c(min(y)-100, max(y) + 100))

**Figure 1:**
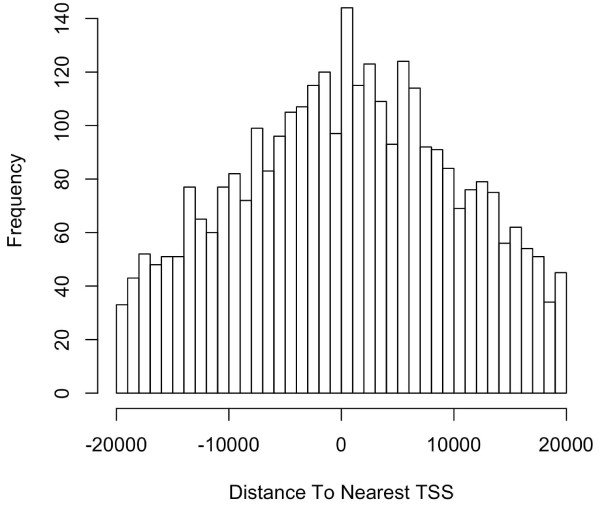
**Distribution of STAT1-binding sites relative to nearest TSSs in human**. The histogram was generated from the putative STAT1-binding regions within 20 kb around TSS identified in un-stimulated cells [2]. The plot shows that STAT1-binding sites are enriched in regions more symmetrically around transcription start sites. The mean distance from the nearest TSS is 8533391 ± 295725 bases (mean ± SEM).

**Figure 2:**
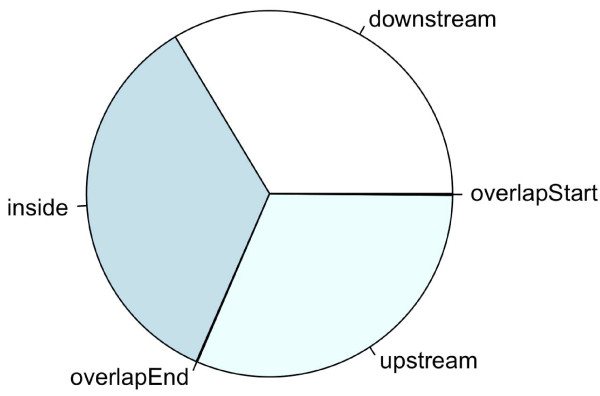
**Pie chart of STAT1-binding sites relative to nearest gene in human**. The pie chart was generated from the putative STAT1-binding regions identified in un-stimulated cells [2]. The plot shows that STAT1-binding sites are evenly distributed along upstream, downstream and inside genes.

Such a plot generated from the putative STAT1-binding regions identified in un-stimulated cells ([2]) shows that the STAT1-binding sites are enriched in regions near TSSs (Figure 1). A pie chart was also generated as follows to show the distribution of relative position of the peaks to the nearest gene (Figure 2).

>temp = as.data.frame(annotatedPeak)

>pie(table(temp [as.character(temp$fromOverlappingOrNearest) == "Overlapping" | (as.character(temp$fromOverlappingOrNearest) == "NearestStart" & !temp$peak %in% temp[as.character(temp$fromOverlappingOrNearest) == "Overlapping",]$peak),]$insideFeature))

It is also possible to obtain the annotation on-line from Ensembl using the *getAnnotation *function as follows prior to calling *annotatePeakInBatch*. Please refer to the *biomaRt *package documentation for a list of available *biomarts *and *datasets *[18].

>mart = useMart(biomart="ensembl", dataset="hsapiens_gene_ensembl")

>Annotation = getAnnotation(mart, featureType="TSS")

To annotate the peaks with other genomic features, it is necessary to change the *featureType *(e.g., "exon" to find the nearest exon, "miRNA" to find the nearest miRNA, "5utr" to find the nearest 5' utr, and "3utr" to find the nearest 3' utr). It is also possible to pass customized annotation data into the function *annotatePeakInBatch*. For example, the user may have a list of transcription factor binding sites from the literature, from a different biological replicate, from a different peak-calling algorithm or from a different protein functioning as transcription complex together with the protein in study, and is interested in determining the extent of the overlap to the list of peaks from his/her experiment. Prior to calling the function *annotatePeakInBatch*, it is necessary to represent both datasets as *RangedData*, where *start *is the start of the binding site, *end *is the end of the binding site, *names *is the name of the binding site, and *space *and *strand *are the chromosome name and strand, respectively, where the binding site is located.

>myexp = RangedData(IRanges(start = c(967654, 2010897, 2496704), end = c(967754, 2010997, 2496804), names = c("Site1", "Site2", "Site3")), space = c("1", "2", "3"))

>literature = RangedData(IRanges(start = c(967659, 2010898, 2496700, 3075866, 3123260), end = c(967869, 2011108, 2496920, 3076166, 3123470), names = c("t1", "t2", "t3", "t4", "t5")), space = c("1", "2", "3", "1", "2"), strand = c(1, 1, -1,-1,1))

>annotatedPeak1 = annotatePeakInBatch(myexp, AnnotationData = literature, output="overlapping", multiple = F, maxgap = 0)

Peaks in text format from peak-calling algorithms can be easily imported to R as *data frame *then converted to *RangedData*. For binding sites represented in BED or GFF format, *BED2RangedData *or *GFF2RangedData *were provided for converting these data formats to *RangedData*.

### Example 2: Determining the significance of the overlapping and visualizing the overlap as a Venn diagram among different datasets

The second example describes how to determine the significance of the overlap, visualize the overlap in a Venn diagram and obtain merged peaks from different datasets such as different biological replicates, different peak-calling algorithms or different proteins functioning as a transcription complex. Here we give examples using different biological replicates.

The first step is to load the *ChIPpeakAnno *package and three example datasets as *RangedData *that contains putative Ste12-binding regions identified in yeast from three biological replicates [31].

>library(ChIPpeakAnno)

>data(Peaks.Ste12.Replicate1)

*>data(Peaks.Ste12.Replicate2*)

*>data(Peaks.Ste12.Replicate3*)

In the next step, the function *makeVennDiagram *is called to generate a Venn diagram to visualize the overlap among the three replicates. In addition, pair-wise overlapping significance tests were performed with hypergeometric test and p-values were generated. *The parameter NameOfPeaks *indicates the names of the dataset for labeling the Venn diagram. *The parameter maxgap *indicates the maximum distance between two peak ranges for them to be considered overlapping. *The parameter totalTest indicates how many potential peaks in total that is used in hypergeometric test*.

>makeVennDiagram(RangedDataList(Peaks.Ste12.Replicate1, Peaks.Ste12.Replicate2, Peaks.Ste12.Replicate3), NameOfPeaks = c("Replicate1","Replicate2","Replicate3"), maxgap = 0, totalTest = 1580)

**Figure 3:**
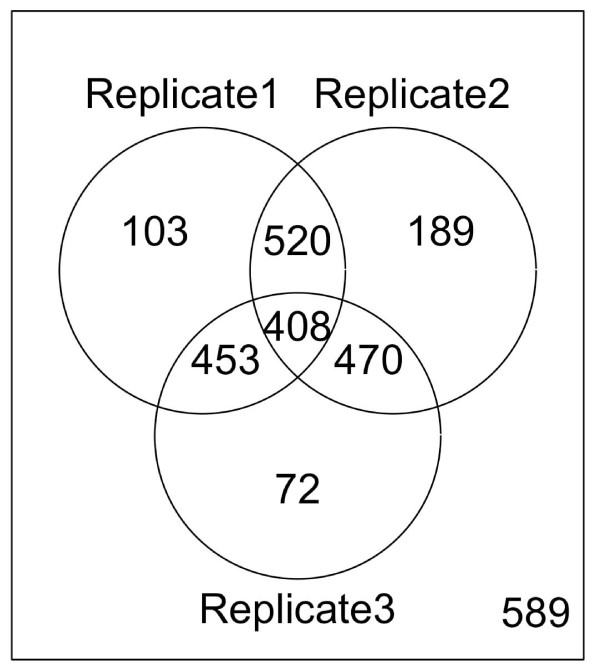
**Overlapping of putative Ste12-binding sites among three biological replicates in yeast**. The Venn diagram was generated from the putative Ste12-binding sites of three biological replicates in yeast [31]. Hypergeometric test shows that there is a significant overlapping among the replicates (p-value < 0.000001 for all three pair-wise comparisons).

**Figure 4:**
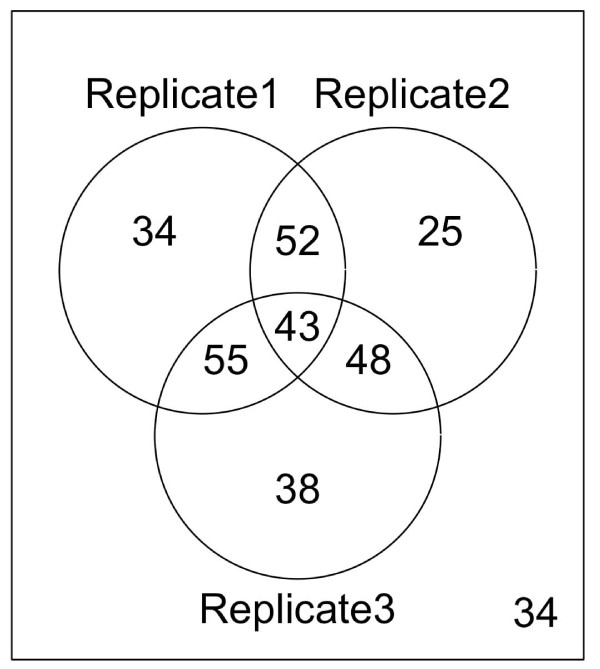
**Overlapping of putative Cse4-binding sites among three biological replicates in yeast**. The Venn diagram was generated from the putative Cse4-binding sites of three biological replicates in yeast [31]. Hypergeometric test shows that the overlapping between biological replicate 1 and 2 is significant at p-value < 0.001 while the other two overlapping are significant at p-value < 0.05.

As a result, a Venn diagram was generated for visualizing the overlap among the above three replicates. The pair-wise overlap comparisons show that the peaks identified from the replicates overlap significantly (Figure 3, p-value < 0.0001). The same analysis was applied to the three biological replicates of Cse4 and the overlap between replicate 1 and 2 is significant at p-value < 0.01 while the other two overlapping is significant at p-value < 0.05 (Figure 4).

The peak ranges from replicates do not overlap perfectly. It is desirable to combine all the overlapping peaks across replicates into merged peaks that cover all the overlapping peaks from the replicates. Calling the function *findOverlappingPeaks *can generate the merged peaks. Besides the parameters mentioned previously, an additional required parameter *multiple *indicates whether to return merged peaks from multiple overlapping peaks.

>MergedPeaks = findOverlappingPeaks(findOverlappingPeaks(Peaks.Ste12.Replicate1, Peaks.Ste12.Replicate2, maxgap = 0, multiple = F, NameOfPeaks1 = "R1", NameOfPeaks2 = "R2")$MergedPeaks, Peaks.Ste12.Replicate3, maxgap = 0, multiple = F, NameOfPeaks1 = "Replicate1Repliate2", NameOfPeaks2 = "R3")$MergedPeaks

**Figure 5:**
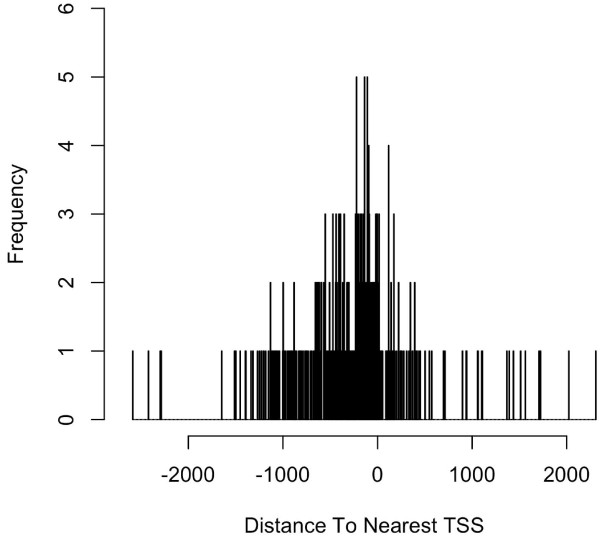
**Distribution of Ste12-binding sites relative to nearest TSSs in yeast**. The histogram was generated from the putative Ste12-binding regions merged from three biological replicates identified in yeast [31]. The plot shows that Ste12-binding sites are enriched in regions upstream and around transcription start sites. The mean of the distance to the nearest TSS is -264 ± 36 bases (mean ± SEM).

**Figure 6:**
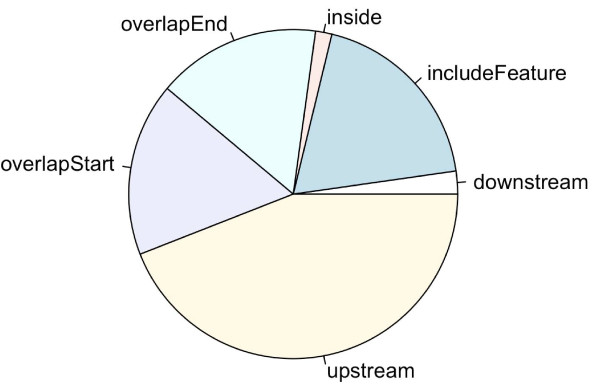
**Pie chart of Ste12-binding sites relative to nearest gene in yeast**. The pie chart was generated from the putative Ste12-binding regions merged from three biological replicates identified in yeast [31] The plot shows that Ste12-binding sites are distributed more on upstream and around the genes.

**Figure 7:**
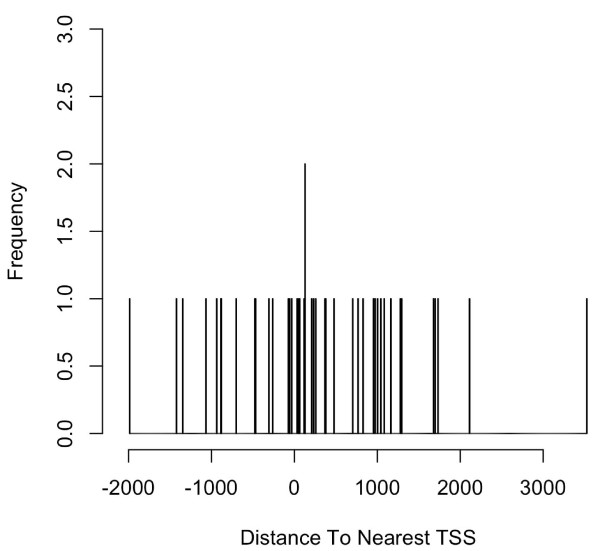
**Distribution of Cse4-binding sites relative to nearest TSSs in yeast**. The histogram was generated from the putative Cse4-binding regions merged from three biological replicates identified in yeast [31]. The plot shows that Cse4-binding sites are enriched in regions inside and downstream of the transcription start sites. The mean of the distance to the nearest TSS is 311 ± 160 bases (mean ± SEM).

**Figure 8:**
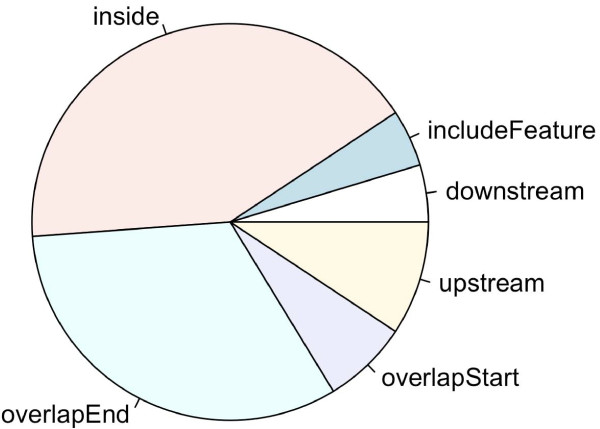
**Pie chart of Cse4-binding sites relative to nearest gene in yeast**. The pie chart was generated from the putative Cse4-binding regions merged from three biological replicates identified in yeast [31]. The plot shows that Cse4-binding sites are distributed more on inside and overlap the end of the genes.

Next, nearest genes and distances between peak location and nearest TSS can be obtained by annotating the merged peaks with SGD1.01 using *annotatePeakInBatch *function illustrated in example 1 (Figure 5 &6). The same analysis was performed with Cse4 binding-sites (Figure 7 &8). The un-equal variance t-test shows that the distribution of the distance of Ste12-binding sites to nearest TSSs (Figure 5, 264 ± 36 bases) is very different from that of Cse4-binding sites (Figure 6, 311 ± 160 bases) (p-value = 0.001). Ste12-binding sites are distributed more towards the upstream of the gene (Figure 5 &6) while Cse4-binding sites are distributed more inside and downstream of the gene (Figure 7 &8). This result is consistent with what has been observed previously [31]. The annotated datasets are available in Additional file 1 and Additional file 2.

### Example 3: Obtaining the sequences around the binding sites for PCR amplification or motif discovery

The third example describes how to obtain the sequences surrounding binding sites (in this example, 100 bp of upstream and downstream sequence) for PCR amplification, cloning or motif discovery [3,30].

The first step is to load the C*hIPpeakAnno *package and create an example peak dataset as *RangedData*. Next, the organism-specific *BSgenome *package is loaded followed by calling the function *getAllPeakSequence*. The function *available.genomes *shows a list of available organism-specific *BSgenome *data packages. In this example, *E. coli *data package is used due its light-weight.

>library(ChIPpeakAnno)

>peaks = RangedData(IRanges(start = c(100, 500), end = c(300, 600), names = c("peak1", "peak2")), space = c("NC_008253", "NC_010468"))

>library(BSgenome.Ecoli.NCBI.20080805)

>peaksWithSequences = getAllPeakSequence(peaks, upstream = 100, downstream = 100, genome = Ecoli)

To convert the sequences to a common FASTA file format, the following function is called.

>write2FASTA(peaksWithSequences, file="test.fa", width = 50)

**Figure 9:**
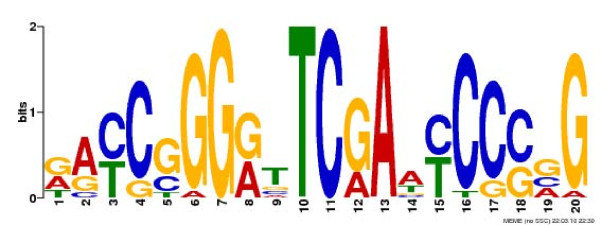
**Motif of Ste12-binding sites in yeast**. The motif was generated using MEME [30] and the sequences from the putative Ste12-binding regions merged from three biological replicates identified in yeast [31]. The default parameters for MEME were chosen except that motif occurrence was set to ZOOP, minimum width to 8, and maximum width to 20.

Sequences for merged Ste12 binding sites were obtained from package *BSgenome.Scerevisiae.UCSC.sacCer2 *(Additional file 3). Significant motifs (E-value < 0.0000001) were identified by running MEME [30] with motif occurrence set as ZOOP, minimum width as 8, maximum width as 20 and other parameters as default (Figure 9).

### Example 4: Obtaining enriched GO terms near the peaks

The fourth example describes how to obtain a list of enriched GO terms associated with adjacent genes using a hypergeometric test.

The first step is to load the TSS annotated peak, which is the result returned from calling the function *annotatePeakInBatch*, and the organism-specific GO gene mapping package (e.g., *org.Hs.eg.db *for the GO gene mapping for human; for other organisms, please refer to http://www.bioconductor.org/packages/release/data/annotation/ for additional org.xx.eg.db packages).

>data(annotatedPeak)

>library(org.Hs.eg.db)

The next step is to call the function *getEnrichedGO*. The parameter *maxP *is the maximum p-value required to be considered to be significant, *multiAdj *indicates whether to apply multiple hypothesis testing adjustment, *minGOterm *is the minimum count in a genome for a GO term to be included, and *multiAdjMethod *is the multiple testing procedure to be applied (for details, see *mt.rawp2adjp *in the *multtest *package).

>enrichedGO <-getEnrichedGO (annotatedPeak [1:6,], orgAnn="org.Hs.eg.db", maxP = 0.01, multiAdj = TRUE, minGOterm = 10, multiAdjMethod="BH")

Where *enrichedGO$bp *contains a list of enriched GO biological process, *enrichedGO$mf *contains a list of enriched GO molecular functions and *enrichedGO$cc *contains a list of enriched GO cellular components.

Table 2 shows a list of enriched GO terms for yeast transcription factor Ste12 [31].

**Table 2:** Enriched GO terms of Ste12-binding sites in yeast.

GO ID	GO Term	GO Definition	Category	FDR
GO:0055114	oxidation reduction	The process of removal or addition of one or more electrons with or without the concomitant removal or addition of a proton or protons.	BP	0.018

GO:0008270	zinc ion binding	Interacting selectively and non-covalently with zinc (Zn) ions.	MF	0.047

GO:0043167	ion binding	Interacting selectively and non-covalently with ions, charged atoms or groups of atoms.	MF	0.046

GO:0043169	cation binding	Interacting selectively and non-covalently with cations, charged atoms or groups of atoms with a net positive charge.	MF	0.046

GO:0043565	sequence-specific DNA binding	Interacting selectively and non-covalently with DNA of a specific nucleotide composition, e.g. GC-rich DNA binding, or with a specific sequence motif or type of DNA e.g. promotor binding or rDNA binding.	MF	0.046

GO:0046914	transition metal ion binding	Interacting selectively and non-covalently with a transition metal ions; a transition metal is an element whose atom has an incomplete d-subshell of extranuclear electrons, or which gives rise to a cation or cations with an incomplete d-subshell. Transition metals often have more than one valency state. Biologically relevant transition metals include vanadium, manganese, iron, copper, cobalt, nickel, molybdenum and silver.	MF	0.046


The enriched GO terms were obtained from the putative Ste12-binding regions merged from three biological replicates identified in yeast [31]. The parameter used to generate the list is *maxP *= 0.05, *multipAdj *= TRUE, *minGOterm *= 5 and *multiAdjMethod *="BH".

## Conclusions

*ChIPpeakAnno *enables batch annotation of binding sites identified from ChIP-seq, ChIP-chip, CAGE or any technology that results in a large number of enriched genomic regions for any species with existing annotation data within the statistical programming environment R. Allowing users to pass their own annotation data such as different ChIP preparation and a dataset from literature, or existing annotation packages, such as *GenomicFeatures *and *BSgenome*, provides flexibility while the tight integration to the *biomaRt *package enables up-to-date annotation retrieval from the BioMart database. The main advantage of *ChIPpeakAnno *is the ability/flexibility to plug in with other annotation packages, ChIP-chip analysis packages, other fast moving deep-sequencing analysis capabilities and infrastructure and statistical analysis tools in Bioconductor. Another advantage of *ChIPpeakAnno *is that it enables comparison between a set of peaks with any annotation feature objects, between two sets of peaks from replicate experiments or transcription factors within a complex and determination of the significance of the overlap.

The *ChIPpeakAnno *package provides documentation in the form of an interactive manual illustrating the usage of individual functions as well as a vignette containing executable code snippets demonstrating a case-oriented help session. The vignette is run at package building and installation time, and thus also serves as a testing suite. Some of the examples described in the paper are also demonstrated in the vignette.

## Availability and requirement

ChIPpeakAnno is an open source software package under the GNU General Public Licence v2.0 and has been contributed to the Bioconductor Project. The software, source code and documentation are available for download from http://www.bioconductor.org or installed from R by typing source http://bioconductor.org/biocLite.R and biocLite("ChIPpeakAnno"). The package has been tested and run on OS X, Windows and various Linux systems. *ChIPpeakAnno *depends on R version 2.10. 0 or later and the following Bioconductor packages: *biomaRt, multtest, IRanges, limma, Biostrings, BSgenome, and GO.db*. In addition, the lightweight organism-specific package *BSgenome.Ecoli.NCBI.20080805 *and *org.Hs.eg.db *were installed during build time for testing the code snippets in the vignette. All these packages can be downloaded from Bioconductor or installed from R using the http://bioconductor.org/biocLite.R script.

## Authors' contributions

LJZ drafted the manuscript. LJZ and HP developed the software package. CG, NDL, MRG, SML and DSL provided scientific advice. DSL performed the MEME analysis. All authors participated in writing and approved the final manuscript.

## Supplementary Material

Additional file 1 **Annotated Ste12-binding sites.** An Excel file contains the annotated Ste12-binding sites merged from the three biological replicates in yeast [31]. Click here for file

Additional file 2 **Annotated Cse4-binding sites. **An Excel file contains the annotated Cse4-binding sites merged from the three biological replicates in yeast [31]. Click here for file

Additional file 3 **Sequence file of Ste12-binding sites used for MEME input. **FASTA formatted sequence file from Ste12-binding sites merged from the three biological replicates in yeast [31]. Click here for file
